# Integrative metabolomic and transcriptomic reveals potential mechanism for promotion of ginsenoside synthesis in Panax ginseng leaves under different light intensities

**DOI:** 10.3389/fbioe.2023.1298501

**Published:** 2023-11-22

**Authors:** Ping Di, Xiao Yang, Mingming Wan, Mei Han, Yonggang Zhang, Limin Yang

**Affiliations:** ^1^ Key Laboratory by Province and the Ministry of Science and Technology of Ecological Restoration and Eco-system Management, College of Chinese Medicinal Material, Jilin Agricultural University, Changchun, China; ^2^ Changchun BCHT Biotechnology Co., Ltd., Changchun, China; ^3^ National Engineering Laboratory for AIDS Vaccine, School of Life Sciences, Jilin University, Changchun, China

**Keywords:** light intensity, Panax ginseng, secondary metabolism, photosynthetic physiology, metabolome

## Abstract

*Panax ginseng* C.A. Meyer is a shade plant, and its leaves are an important medicinal part of *P. ginseng*. Light intensity plays a crucial role in physiological activities and metabolite accumulation in *P. ginseng*. Currently, little is known about the molecular mechanisms underlying physiological changes and quality under different light intensities in *P. ginseng* leaves. Therefore, we investigated the changes in photosynthetic physiology, secondary metabolism, transcriptomics, and metabolomics of *P. ginseng* leaves under different light intensities [T20 (20 µmol m^-2^·s^-1^), T50 (50 µmol m^-2^·s^−1^), T100 (100 μmol m^−2^·s^−1^)]]. Higher light intensity positively influenced the yield, photosynthesis, and accumulation of polysaccharides, soluble sugars, terpenoids, and ginsenosides in *P. ginseng* leaves. The T100 treatment notably promoted the accumulation of ginsenosides in the leaves, resulting in a 68.32% and 45.55% increase in total ginsenosides compared to the T20 and T50 treatments, respectively. Ginsenosides Rg1, Re, Rb1, Rc, Rg2, Rb2, Rb3, and Rd were 1.28-, 1.47-, 2.32-, 1.64-, 1.28-, 2.59-, 1.66-, and 2.28-times higher than in the T20 treatment. Furthermore, 285 differentially accumulated metabolites (DAMs) and 4218 differentially expressed genes (DEGs) in the metabolome and transcriptome of *P. ginseng* leaves, respectively, were identified. 13 triterpenoid saponins were significantly upregulated, and three were downregulated. The expression of genes encoding photosystem II reaction center proteins was upregulated under the T100 treatment, thereby increasing photosynthetic activity. The T100 treatment enhanced the expression of genes involved in photosynthetic carbon and energy metabolism in *P. ginseng*. The expression of antenna protein synthesis genes was upregulated under the T20, which increased the ability to capture light in *P. ginseng* leaves. T100 upregulated the expression of HMGR, SS, CYP716A53v2, UGT74AE, PgUGT1, and UGTPg45, thereby promoting terpene and ginsenoside synthesis. In summary, 100 µmol m^−2^·s^−1^ was conducive to quality formation of *P. ginseng* leaves. This study elucidates molecular mechanisms underlying the photosynthetic physiology and ginsenoside synthesis in *P. ginseng* under varying light intensities and provides a theoretical basis for the *P. ginseng* cultivation and its industrial production of secondary metabolites.

## 1 Introduction

*Panax ginseng* C.A. Meyer is a perennial herb belonging to the Araliaceae family and has substantial medicinal and economic value. Research has demonstrated that ginsenosides exhibit a diverse range of pharmacological activities. For example, ginsenoside Rg_1_ can reduce oxidative damage in the liver (Gao et al., 2017). Ginsenoside Re is effective against diabetes mellitus, nervous system diseases, cardiovascular disease, and cancer (Gao X. Y. et al., 2022). Various ginsenosides exhibit anti-inflammatory, antioxidant, antibacterial, and antiviral properties (Huang et al., 2021; Fan et al., 2023). *Panax ginseng* grows in the understory of deciduous or mixed coniferous broad-leaved forests at altitudes ranging in the hundreds of meters. The field cultivation of *P. ginseng* requires shade shelters to regulate the light environment. With advances in technology, artificial light sources have been considered a viable option for regulating light intensity in *P. ginseng* cultivation, providing an opportunity to elucidate the physiological responses and metabolic processes of *P. ginseng* under different light conditions. It also presents possibilities for the implementation of novel cultivation models, such as plant factories, for *P. ginseng* production.

*P. ginseng* is born in deciduous broad-leaved forests or mixed coniferous broad-leaved forests at an altitude of several hundred meters. In field cultivation, it is common to control the light intensity of *P. ginseng* by building a shade shelter to simulate the growth conditions of *P. ginseng*. The regulation of light intensity is important for the yield and quality of *P. ginseng.* Previous studies have investigated the optimal light intensity for *P. ginseng* growth. Jang (Jang et al., 2021) reported that the range of 75 µmol m^−2^·s^−1^–100 µmol m^−2^·s^−1^ was suitable for *P. ginseng* growth. Lee’s study indicated that a light intensity of 50 µmol m^−2^·s^−1^ accompanied by a 12 h d^−1^ photoperiod was advantageous for the growth of shoots and roots in *P. ginseng* (Lee et al., 2022). However, none of these studies have extensively investigated the mechanisms governing the physiology and secondary metabolism of *P. ginseng* under different light intensities. Both the roots and leaves are medicinal parts of *P. ginseng*. The Chinese Pharmacopoeia stipulates that *P. ginseng* leaves are also a part of the medicine, and the leaves are rich in ginsenosides. However, most of these studies have focused on roots. *Panax ginseng* leaves also have important medicinal and economic properties. Usually, *P. ginseng* roots can be harvested in 4–6 years, but the leaf time is 1 year. The saponin content of leaves is much higher than that of the roots (Lee et al., 2017). China produces approximately 20.0 Mt of stem leaves annually, which are primarily used for extracting saponin (Zhang F. et al., 2021). Research on *P. ginseng* leaves is still required, which has resulted in the economic value of leaves being ignored and a large number of stem leaves being discarded. Conducting basic and applied research on the pharmacodynamically active components of leaves is conducive to the optimal use of resources.

Light is an important source of energy for plants. Through photosynthesis, green plants convert solar energy into chemical energy to sustain the Earth’s ecosystems. Light is crucial for all life on Earth. Photosynthesis involves a series of electron transfer processes through photosystems I and II, which ultimately results in photosynthetic carbon fixation (Allen, 2018). In nature, the light environment of plants is complex. The light quality and intensity of leaves undergo constant dynamic changes due to factors such as diurnal variations, cloud cover, movement of light spots, and shading from the canopy (Fu and Walker, 2023). Plants exhibit diverse responses to light during their physiological processes to adapt to complex light environments. For instance, plants enhance their capacity for light capture, improve photochemical efficiency, and may even develop shade characteristics by increasing internode length and leaf area to capture more light energy under low-light conditions (Critchley, 1981). However, under strong light conditions, plants reduce their light capture capacity and dissipate excessive light energy for photoprotection (Zhang et al., 2017). Light intensity affects various physiological and ecological processes in plants, including morphological characteristics, photosynthesis, antioxidant enzyme systems, and secondary metabolism.

Secondary metabolites are the main active ingredients of medicinal plants and have a variety of pharmacological effects and are widely used in the medical and food fields (Linlin et al., 2022). The synthesis and accumulation of active constituents in medicinal plants are regulated by light intensity. Currently, at least 25% of medicines globally are directly or indirectly derived from plants (Ansari et al., 2013). Therefore, it is important to determine optimal light intensity conditions for medicinal plants to improve their quality and is an important part of the plant factory production mode. The main active components of medicinal plants are secondary metabolites with complex biosynthetic processes. Ginsenosides are classified as protopanaxadiol (PPD)-, protopanaxatriol (PPT)-, and oleanane-type saponins (OTS). Terpenoid biosynthesis occurs primarily via the mevalonate (MVA) and methylerythritol-4-phosphate (MEP) pathways, both starting from acetyl coenzyme A. During the synthesis process of *P. ginseng*, HMGR, FPS, SS, SE, and DS play important roles in the synthesis of the triterpene skeleton, whereas cytochrome P450s (CYPs) and glycosyltransferases (UGTs) complete the final modification of the ginsenoside structure. Currently, the ginsenoside synthesis pathway is relatively clear; however, studies on how light intensity regulates the expression of ginsenoside synthesis genes are lacking.

Leaves are the primary organs involved in photosynthesis, which contain large number of secondary metabolites. Currently, research on light control in *P. ginseng* leaves is still not systematic, and the related physiological and secondary metabolic changes remain vague. Therefore, we performed a systematic analysis of photosynthetic physiology, transcriptomics, and metabolomics. We analyzed the changes in transcripts and metabolites in *P. ginseng* leaf samples using transcriptome and metabolome techniques and interpreted the physiological adaptations to different light intensities. In addition, our study elucidated the molecular mechanisms underlying ginsenoside synthesis and accumulation in response to light intensity. These findings will provide new insights into the response mechanisms and physiological changes in *P. ginseng* plants under different light intensities and provide references for the *P. ginseng* cultivation and its industrial production of secondary metabolites. It also promoted the technological innovation of *P. ginseng* cultivation and the application of modern engineering technology in the field of ginseng cultivation.

## 2 Materials and methods

### 2.1 Plant materials and light treatments

The experimental site was located at the agricultural facility of the Jilin Agricultural University, China. We used 3-year-old *P. ginseng* seedlings that were obtained from Jilin Shenyang and Plant Protection Technology Co., Ltd. (Fusong, China). The cultivated soil was a mixed substrate of the amended soil, vermiculite, and perlite (7:3:1). The physical and chemical properties of the soil were pH 5.56, 1.32 mg/kg organic carbon, 124.34 mg/kg available nitrogen, 13.54 mg/kg available phosphorus, and 164.23 mg/kg available potassium. On 30 October 2020, *P. ginseng* seedlings with the same growth status and well-developed roots were selected. Seedlings were treated with 100 ppm gibberellin for 12 h and then transplanted into polypropylene pots [30.5 cm (diameter) × 28.5 cm (height)]. Each pot was filled with soil of the same quality. Three *P. ginseng* plants were transplanted into each pot. On 4 December 2020, *P. ginseng* seedlings with normal growth and development were used for the light-intensity treatments. The experiment included three light intensity levels: 20 µmol m^−2^·s^−1^ (T20), 50 µmol m^−2^·s^−1^ (T50), and 100 µmol m^−2^·s^−1^ (T100). Natural light (NL) treatment in the greenhouse was used as a control. In the culture room, cool-white, fluorescent lamps (OPPLE, 28 W/6500 K, Shanghai, China) were used to regulate the light intensity. The light intensity above the *P. ginseng* leaves was measured using a quantum sensor (ADC BioScientific Co., Ltd., United Kingdom) and the final light intensity value for the treatment was determined by calculating the average of eight points. The light period was set from 06:00 to 18:00 (12 h/12 h). The temperature was set at 25°C/20°C. Routine management practices were implemented during the light-intensity experiments. Samples were collected at 30, 60, and 100 days. We selected fifteen *P. ginseng* plants and carefully cleaned to measure their growth indices. A part of the fresh samples was promptly frozen in liquid nitrogen and kept at −80°C. The remaining samples were dried at 50°C.

### 2.2 Determination of photosynthetic and chlorophyll fluorescence parameters

An LCpro + portable photosynthesis system (LCpro+, ADC BioScientific Co., Ltd., United Kingdom) was used to determine the photosynthetic parameters. We chose five *P. ginseng* plants and three of the largest leaves from each plant were chosen to measure the following parameters: photosynthetic rate (Pn, µmol·m^−2^·s^−1^), stomatal conductance (Gs, mmol·m^−2^·s^−1^), atmospheric CO_2_ concentration (Ca, µmol·mol^−1^), transpiration rate (Tr, mmol H_2_O·m^−2^·s^−1^), and intercellular CO_2_ concentration (Ci, µmol·mol^−1^). The leaves were fully extended in a 6.25 cm^2^ leaf chamber. Data were collected when the CO_2_ assimilation rate reached a steady state. Stomatal limitation values (Ls = 1-Ci/Ca) and instantaneous water-use efficiency (WUE = Pn/Tr) were calculated. Chlorophyll fluorescence parameters were measured using a chlorophyll fluorescence instrument (OS-5P+; Opti-Sciences, Co., Ltd., United States). The leaves were dark-adapted for 30 min prior to measurements. The initial fluorescence (Fo), maximum fluorescence (Fm), steady-state fluorescence (F ′), minimum fluorescence under light (Fo′), and maximum fluorescence under light (Fm′) were measured. The following equations were used: maximum photochemical efficiency, Fv/Fm = (Fm-Fo)/Fm), actual photochemical efficiency, φPSII = (Fm′-F′)/Fm′), photochemical quenching coefficient, qP = (Fm′-F′)/(Fm′-Fo′), and non-photochemical quenching coefficient, NPQ = (Fm-Fm′)/Fm′ for photosystem II (PSII) (Yang et al., 2019).

### 2.3 Determination of content of soluble sugar, soluble protein and soluble polysaccharide

An amount of 0.5 g of fresh tissue and 4.5 mL PBS (0.05 mol/L) were mixed and ground. The tissue homogenate was centrifuged for 10 min (4,500 rpm/min) in an ice water bath, and the supernatant was collected for testing. The tissue homogenates was treated with soluble sugar and protein kits (Nanjing Jiancheng Bioengineering Institute, NJBI, China). The absorbance of the reaction solution was measured at 620 and 595 nm using an enzyme-labeling instrument to determine the soluble sugar and protein content (Spectra Max 190, Molecular Devices, United States). The soluble sugar and protein contents were calculated according to the manufacturer’s instructions.

In this study, a hot water extraction method was used to extract the polysaccharides (He et al., 2020). An amount of 0.5 g of *P. ginseng* sample was added to 25 mL distilled water (material-to-liquor ratio of 1:50). The mixture was extracted in a water bath at 100°C for 4 h and then filtered and centrifuged. Ethanol (80%) was then added to the supernatant liquid. After refrigerating at 4°C overnight, the extract was centrifuged at 5,000 rpm for 10 min. The precipitate was dissolved in hot distilled water to obtain the polysaccharide extract from the sample. The phenol-sulfuric acid method was used to determine the polysaccharides (DuBois et al., 1956). To establish the standard solution for polysaccharide determination, 10 mg of a glucose standard was accurately weighed and dissolved in a 10 mL volumetric flask (1 mg/mL). Different volumes (0, 40, 80, 120, 160, and 200 µL) of the glucose standard solution were added to 2 mL of distilled water. A 5% phenol solution and 5 mL of H_2_SO_4_ was added to the glucose solution. The solution was then left at room temperature for 5 min and subjected to a 15 min boiling water bath. Finally, the sample was rapidly cooled in an ice-water bath. The absorbance was measured at 485 nm using an Evolution 201 UV spectrophotometer (Thermo Scientific, United States). A standard curve of glucose concentration and absorbance was plotted to obtain a regression equation (y = 0.0124*x* + 0.0856, *R* ^2^ = 0.9992). Polysaccharides from *P. ginseng* leaves, stems, and roots were extracted and analyzed using the aforementioned procedure.

### 2.4 Determination of content of ginsenosides

Ginsenosides were extracted using ultrasonic extraction. One Gram of *P. ginseng* powder was added to 30 mL methanol, conducted for 30 min at 30°C at an ultrasonic frequency of 40 kHz. This process was repeated three times, and the extract solution was filtered, and the volume was reduced to 5 mL. An 0.22-µm organic phase filter was used for filter extraction. An HPLC system (1260 Infinity II, Agilent, United States) was used to determine the ginsenosides following the method described by Tao et al. (Zhang T. et al., 2021). We used a reversed-phase column (ZORBAX SB-C18, 4.6 mm × 250 mm, 5 µm Agilent, United States) for the determination. The mobile phase consisted of 100% water (A) and 100% acetonitrile (C). The flow rate was set at 0.8 mL/min, and the detection wavelength was 203 nm. The column temperature was 25°C, and the injection volume was 10 µL. The gradient program was as follows:0–36 min, 18%–21% C; 37–41 min, 21%–28% C; 41–45 min, 28%–34% C; 45–54 min, 34%–38% C; 54–61 min, 38%–71% C; 61–80 min, 71%–80%, 80–100 min, 80%–18% C. Commercial-grade ginsenosides Rg1 (G16S10Y97436), Re (B04D9576499), Rf (P13S9L70209), Rb1 (Z20S9X70603), Rb2 (P15O10F94983), Rc (M15O10S100110), Rg2 (80952-72-3), Rd (Z13N8X48155), and Rb3 (Y05A8Y41182) were purchased from Shanghai Source Leaf Biological Technology Co., Ltd. and the concentration ≧98%. The resulting standard curve equations were:

$$\text{Rg}1\left(y=471.1x+7.3252\right),$$ (1)

$$\text{Re}\left(y=385.02x+5.7274\right),$$ (2)

$$\text{Rf}\left(y=357.2x+0.9383\right),$$ (3)

$$\text{Rb}1\left(y=276.14x+5.6388\right),$$ (4)

$$\text{Rc}\left(y=239.24x+5.5644\right),$$ (5)

$$\text{Rg}2\left(y=450.48x+2.9523\right),$$ (6)

$$\text{Rb}2\left(y=269.98x+3.0672\right),$$ (7)

$$\text{Rb}3\left(y=269.55x+0.7054\right),$$ (8)

and

$$\text{Rd}\left(y=296.91x+0.9281\right).$$ (9)

### 2.5 Metabolite profiling analysis

Leaf samples of *P. ginseng* were freeze-dried in a vacuum freeze dryer (Scientz-100F). The freeze-dried samples were pulverized and then dissolved. The UPLC-ESI-MS/MS system (UPLC, SHIMADZU Nexera X2, Applied Biosystems 4500Q TRAP) was used to analyze the sample extracts. The conditions were set as: UPLC column-Agilent SB-C18 (1.8µm, 2.1 mm × 100 mm); mobile phase - Solvent A (pure water + 0.1% formic acid) and Solvent B (acetonitrile + 0.1% formic acid). The sample measurements were performed using a gradient program. The initial conditions were 95% solvent A and 5% solvent B. A linear gradient was applied for 9 min starting with 5% A and 95% B solutions. The 5% A and 95% B components were maintained for 1 min, followed by a 1.1-min adjustment to the 95% A and 5% B components, which were then held for 2.9 min. The flow rate was set to 0.35 mL/min, the column oven temperature was maintained at 40°C, and the injection volume was 4 μL. The effluent was then directed to an ESI-triple quadrupole linear ion trap (QTRAP)MS instrument.

LIT and triple quadrupole (QQQ) scans were acquired using an AB4500 Q TRAP UPLC-MS/MS system, which is a triple quadrupole linear ion-trap mass spectrometer. The system was equipped with an ESI turbo ion–spray interface and operated in both positive and negative ion modes. The instrument was controlled using Analyst 1.6.3 software (AB Sciex). The ESI source operation parameters were as follows: ion source—turbo spray; source temperature—550°C; ion spray voltage (IS)—5,500 V (positive ion mode), and −4,500 V (negative ion mode); ion source gas I (GSI), gas II (GSII), and curtain gas (CUR) were set at 50, 60, and 25.0 psi, respectively; collision-activated dissociation (CAD) was set to high. QQQ scans were acquired in the MRM experiments using medium collision gas (nitrogen) settings. A specific set of MRM transitions was monitored for each period, based on the eluted metabolites.

### 2.6 Transcriptome analysis

Total RNA of *P. ginseng* leaf samples was extracted using a Trizol kit (Invitrogen, CA, United States), and RNA purity (OD260/280 1.8-2.2) was measured using a NanoPhotometer spectrophotometer (IMPLEN, CA, United States). RNA concentration was accurately determined using a Qubit 2.0 fluorometer (Life Technologies, Carlsbad, CA, United States), and RNA integrity was precisely assessed using an Agilent 2100 Bioanalyzer (Agilent Technologies, CA, United States). The quality criteria of total RNA were:28S/18S ≥ 1.0, total RNA content ≥ 2.0 μg, total RNA concentration ≥ 50 ng-μL-1, RIN > 7.0. A cDNA library was constructed from high-quality total RNA. The Illumina NovaSeq 6000 (LC Sciences, TX, United States) platform was used for sequencing. The image data CASAVA base identification were converted into a large amount of high-quality data (Raw Data). The read data were subjected to rigorous quality control using FASTP software before data analysis to ensure the accuracy of subsequent analyses. This quality control step involved the removal of reads with adapters, paired reads with high N content, and paired reads with low-quality bases (Q ≤ 20). The remaining clean reads used for subsequent analysis were obtained after filtering the raw data, checking the sequencing error rate, and examining the GC content distribution.

Clean Reads of *P. ginseng* samples were aligned to the reference genome using HISAT2 (Kim et al., 2015). The *P. ginseng* genome data can be found at (https://ngdc.cncb.ac.cn/gwh/Assembly/22230/show). The sequences were aligned to individual exons of the genome or segmented alignments were performed for two or more exons, including three or more exons. To obtain gene annotation information, the gene sequences were aligned to the NR, Swiss-Prot, GO, and KEGG databases using DIAMOND BLASTX software (Buchfink et al., 2015), while the amino acid sequences of the genes were aligned to the Pfam database using HMMER software. Reads per kilobase per million reads (FPKM) were used to screen genes in the transcriptome data. Differentially expressed genes (DEGs) were screened according to *p* < 0.05 and |log_2_ (fold change)|≥1. To elucidate the functions and biological roles of the DEGs, the DEGs obtained from different treatment groups we mapped to terms in the GO database. The number of genes associated with each GO function was determined. To investigate the metabolic pathways associated with DEGs and elucidate the biological processes underlying gene–gene interactions, enrichment analysis of the DEGs in each KEGG pathway was conducted.

### 2.7 Statistical analysis

Original data were processed using Excel 2019 (Microsoft Corp., Redmond, WA, United States). All statistical analyses were performed using SPSS version 19 (IBM Corp., Armonk, NY, United States). GraphPad Prism 6 (GraphPad Software Inc., San Diego, CA, United States) was used to generate graphics. OmicStudio tools (https://www.omicstudio.cn/tool) were used for bioinformatics analysis and graphics.

## 3 Results

### 3.1 Effect of light intensities on the growth of P. ginseng

**FIGURE 1:**
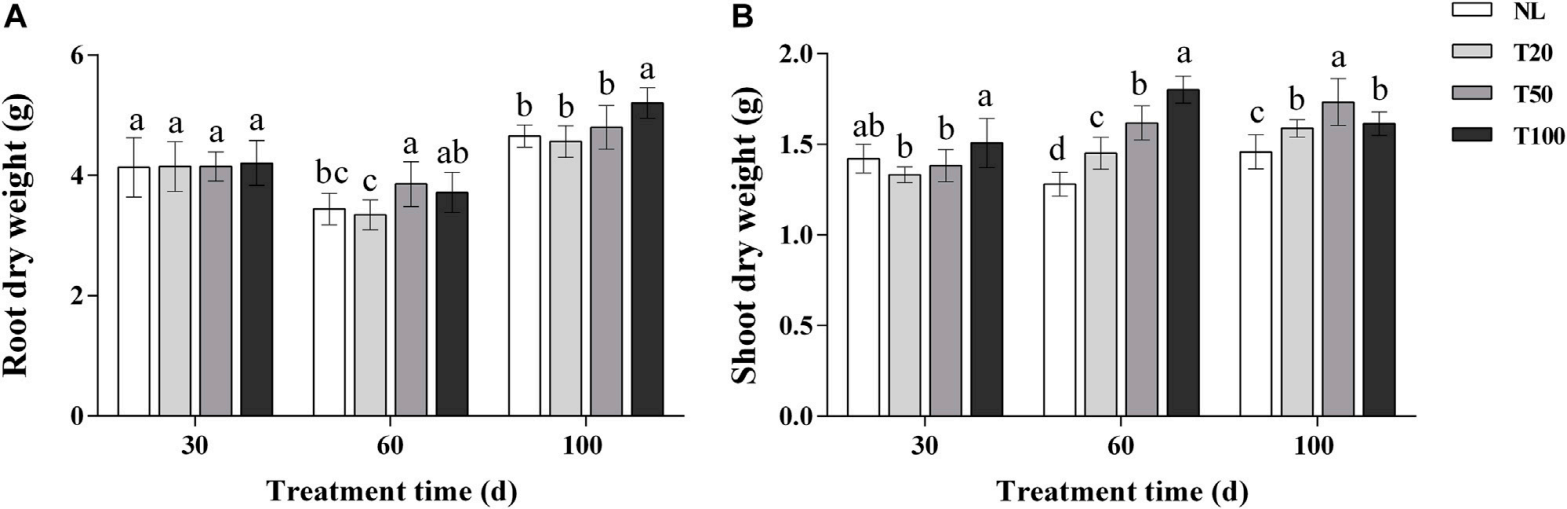
Effect of different light intensities on root dry weight **(A)** and shoot dry weight **(B)**. Note: NL, natural light treatment; T20, 20 µmol m^−2^·s^−1^ light intensity; T50, 50 µmol m^−2^·s^−1^ light intensity; T100, 100 µmol m^−2^·s^−1^ light intensity. The data are expressed as the mean ± standard deviation (SD) (Duncan, *p* < 0.05).

The effects of different light intensities on the dry weight of ginseng roots and the dry weight of aboveground parts are shown in Figure 1. At 30 days of light intensity treatment, there was no significant difference in the dry weight of ginseng roots, and the aboveground dry weight of T100 treatment was significantly higher than that of T20 treatment. At 60 days of light intensity treatment, the aboveground dry weight increased with the increase of light intensity, and the aboveground dry weight of T100 treatment increased by 24.20% compared with that of T20 treatment, and increased by 40.61% compared with that of NL treatment. Root dry weight reached its maximum in T50 treatment and increased by 15.36% compared with T20 treatment. At 100 days of light intensity treatment, ginseng root dry weight reached maximum under T100 treatment and increased by 14.00% compared to T20 treatment. Above ground dry weight reached maximum at T50 treatment and increased by 18.89% compared to NL treatment.

### 3.2 Effect of light intensities on photosynthetic and chlorophyll fluorescence parameters of P. ginseng

**FIGURE 2:**
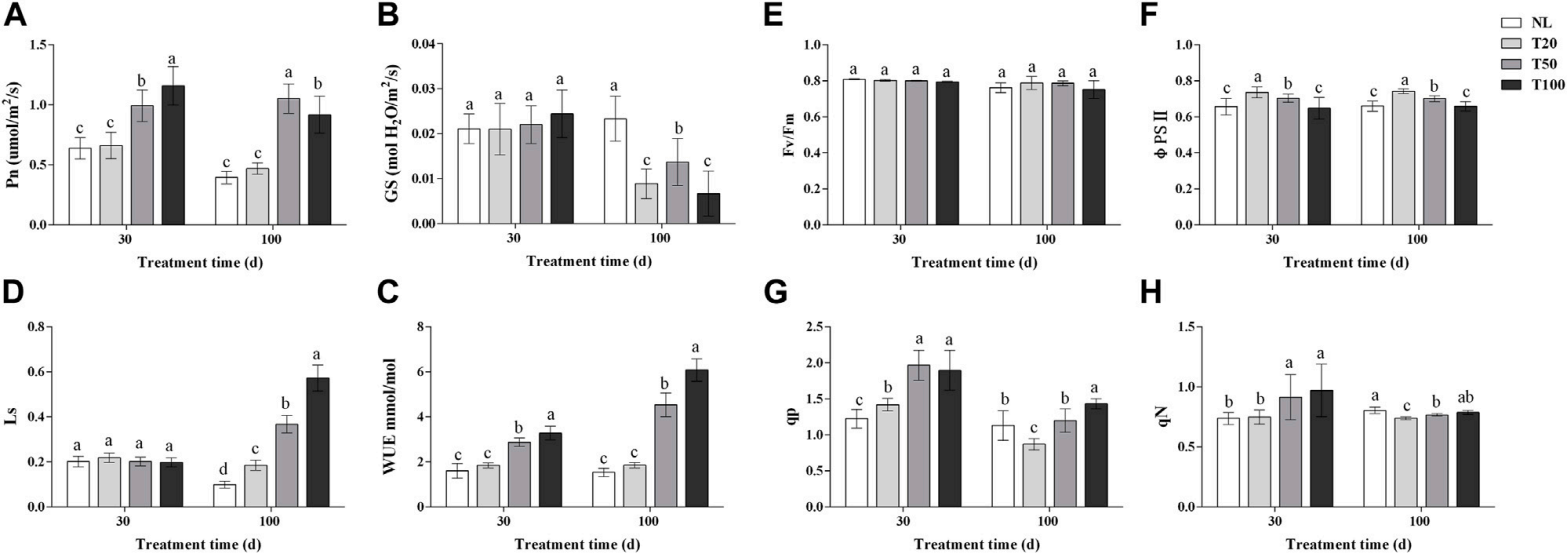
Photosynthetic and chlorophyll fluorescence parameters under different light intensities **(A)**, photosynthesis rate (Pn); **(B)**, water-use efficiency (WUE); **(C)**, stomatal conductance (Gs); **(D)**, stomatal limit value (Ls); **(E)**, photochemical efficiency (*Fv/Fm*); **(F)**, actual photochemical efficiency (φPSⅡ); **(G)**, photochemical quenching coefficient (qp); **(H)**, non-photochemical quenching coefficient (NPQ). Note: NL, natural light treatment; T20, 20 µmol m^−2^·s^−1^ light intensity; T50, 50 µmol m^−2^·s^−1^ light intensity; T100, 100 µmol m^−2^·s^−1^ light intensity. The data are expressed as the mean ± standard deviation (SD) (Duncan, *p* < 0.05)

Light intensity changed the photosynthetic characteristics and chlorophyll fluorescence parameters of *P. ginseng* (Figure 2). After 30 days of light treatment, the Pn of *P. ginseng* leaves increased with increasing light intensity. The Pn of the T100 treatment was 1.15 times higher than that of the T20 treatment, whereas no significant difference was observed between the T20 and NL treatments. Pn was the highest under the T50 treatment after 100 days of light treatment. The Gs of *P. ginseng* leaves under different light intensities showed no difference after 30 days. However, at 100 days, the Gs of the NL treatment was significantly higher than that of the other treatments (*p* < 0.05). Both the T20 and T100 treatments had an inhibitory effect on stomatal opening in *P. ginseng* leaves, resulting in decreases of 35.04% and 51.09%, respectively, compared to the T50 treatment. Moreover, both the T50 and T100 treatments markedly increased the WUE of the leaves compared to the T20 treatment. After 100 days of light intensity treatment, the Ls values followed the order: T100 > T50 > T20 > NL.

After 30 days of light treatment, there was no difference in fv/fm among the treatments. The order of φPSII was T20 > T50 > T100, with the T20 treatment showing an increase of 11.95% compared to the NL treatment, while T100 treatment was similar to the NL treatment. Both the T50 and T100 treatments significantly increased qp in *P. ginseng* leaves by 38.70% and 33.74%, respectively, compared to the T20 treatment (*p* < 0.05). Similarly, qN was significantly higher in both the T50 and T100 treatments than in the T20 treatment, with increases of 22.12% and 29.75%, respectively (*p* < 0.05). After 100 days of light treatment, there was no statistical difference in fv/fm among the treatments, but it exhibited a decreasing trend with increasing light intensity. In terms of PSII, a negative correlation was observed with an increase in light intensity. The order of qp was T20 > T50 > T100, and qp under the T100 and T50 treatments increased by 37.81% and 33.73%, respectively, compared to the T20 treatment. The qN of *P. ginseng* leaves increased with increasing light intensity.

### 3.3 Effects of different light intensity on the contents of soluble sugar and protein in P. ginseng

**FIGURE 3:**
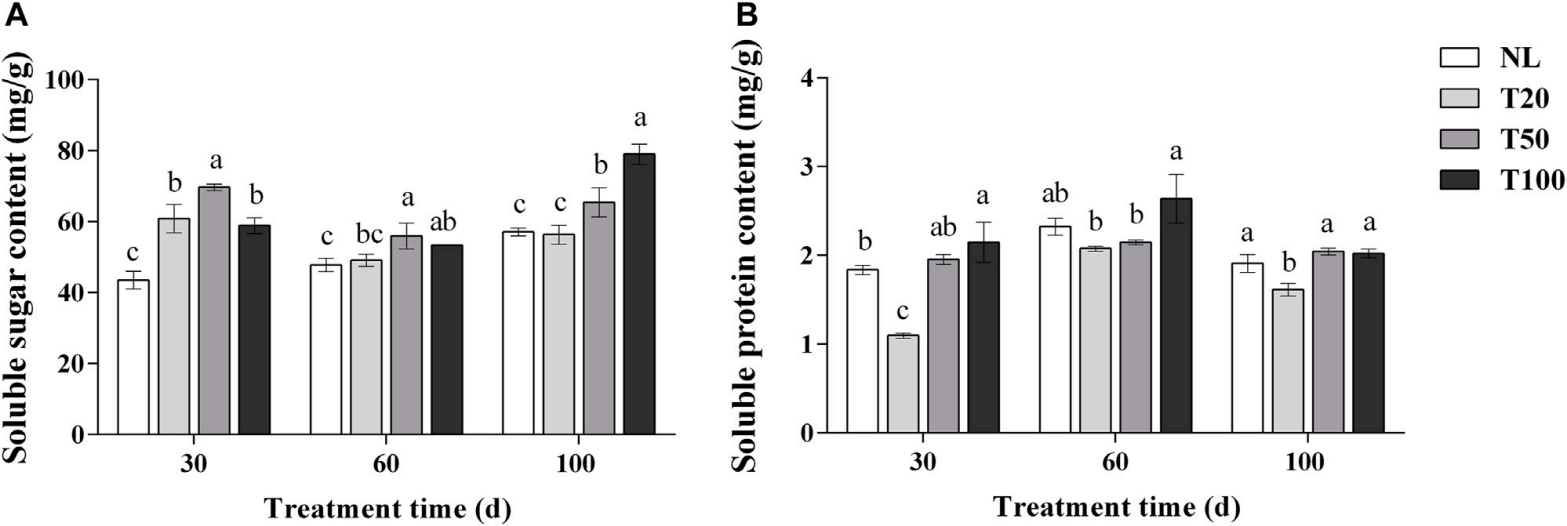
Effect of different light intensities on the content of soluble sugar **(A)** and soluble protein **(B)**. Note: NL, natural light treatment; T20, 20 µmol m^−2^·s^−1^ light intensity; T50, 50 µmol m^−2^·s^−1^ light intensity; T100, 100 µmol m^−2^·s^−1^ light intensity. The data are expressed as the mean ± standard deviation (SD) (Duncan, *p* < 0.05)

The effects of different light intensities on soluble sugar and protein contents are shown in Figure 3. After 30 days of light intensity treatment, the T50 treatment exhibited a significant increase in the soluble sugar content of *P. ginseng* leaves to 69.70 mg/g, which was 14.47% higher than that of the T20 treatment (*p* < 0.05). The soluble sugar content in the T20, T50, and T100 treatments was significantly higher than that in the NL treatment. Similar trends were observed in the soluble sugar content of *P. ginseng* leaves after 60 days of light treatment. At 100 days, the content of soluble sugar content reached 79.08 mg/g under the T100 treatment, which was 38.40%, 40.18%, and 20.77% higher than the NL, T20, and T50 treatments, respectively. The soluble protein content in *P. ginseng* leaves increased with increasing light intensity after 30 days of treatment. The soluble protein content under NL, T50, and T100 treatments was higher than that of T20 treatment, which increased by 18.39%, 26.65%, and 25.30%, respectively, compared to the T20 treatment. The same trend was observed after 100 days of light treatment, but the difference in content between the T20 treatment and each treatment became less pronounced.

### 3.4 Effect of light intensity on polysaccharide content of root, stem, and leaf of P. ginseng

Light intensity treatment altered the polysaccharide content of *P. ginseng* (Table 1). After 60 days of light intensity treatment, the polysaccharide contents of the T50 and T100 treatments were 9.03% and 9.41%, respectively, which were 28.56% and 23.36% higher than those of the T20 treatment, respectively. After 100 days of light intensity treatment, the polysaccharide content in ginseng roots followed the order T100 > T50 > T20, and the polysaccharides of *P. ginseng* roots in the T100 and T50 treatments increased by 52.80% and 18.94%, respectively, compared to the NL treatment. After 30 days of light intensity treatment, the highest polysaccharide content was observed in the T100 treatment of the stems, while the opposite trend was observed at 60 days, indicating that the polysaccharide content was the lowest in T100. Similarly, the polysaccharide content in the leaves at 30 days was similar to that in the stems, and the polysaccharide content in the T100 treatment reached 1.87%, which was 23.84% higher than that of natural light (NL) and 25.50% higher than that of the T50 treatment. At day 60, the polysaccharide contents in the NL and T20 treatments substantially increased. These results demonstrate that high light intensity treatment positively influence the soluble polysaccharide content in *P. ginseng roots.* In addition, the polysaccharide content of stems and leaves varied with different growth periods, which may be associated with the consumption and transport of sugar in *P. ginseng*.

**TABLE 1:** Polysaccharide contents in roots, stems, and leaves of *Panax* ginseng Note: NL, natural light treatment; T20, 20 µmol m^−2^·s^−1^ light intensity; T50, 50 µmol m^−2^·s^−1^ light intensity; T100, 100 µmol m^−2^·s^−1^ light intensity. The data are expressed as the mean ± standard deviation (SD) (Duncan, *p* < 0.05).

	Treatment	Treatment time		
		30 (d)	60 (d)	100 (d)
Root	NL	9.91 ± 0.74a	7.58 ± 0.41b	5.70 ± 0.18c
	T20	8.36 ± 1.21a	7.32 ± 1.21b	5.50 ± 0.71c
	T50	8.02 ± 0.64a	9.03 ± 0.30a	6.78 ± 0.24b
	T100	9.29 ± 1.20a	9.41 ± 0.47a	8.71 ± 0.67a
Stem	NL	1.29 ± 0.08c	1.70 ± 0.13c	1.51 ± 0.16c
	T20	1.50 ± 0.09b	2.43 ± 0.04a	1.18 ± 0.07d
	T50	1.52 ± 0.07b	2.06 ± 0.17b	2.46 ± 0.17a
	T100	1.67 ± 0.03a	1.70 ± 0.11c	1.88 ± 0.12b
Leaf	NL	1.51 ± 0.11b	3.87 ± 0.56a	2.14 ± 0.08a
	T20	1.63 ± 0.12b	3.95 ± 0.48a	2.15 ± 0.12a
	T50	1.49 ± 0.10b	4.25 ± 0.32a	1.91 ± 0.08b
	T100	1.87 ± 0.13a	3.61 ± 0.02a	1.91 ± 0.06b

### 3.5 Effect of different light intensities on the ginsenoside content of P. ginseng leaves

**FIGURE 4:**
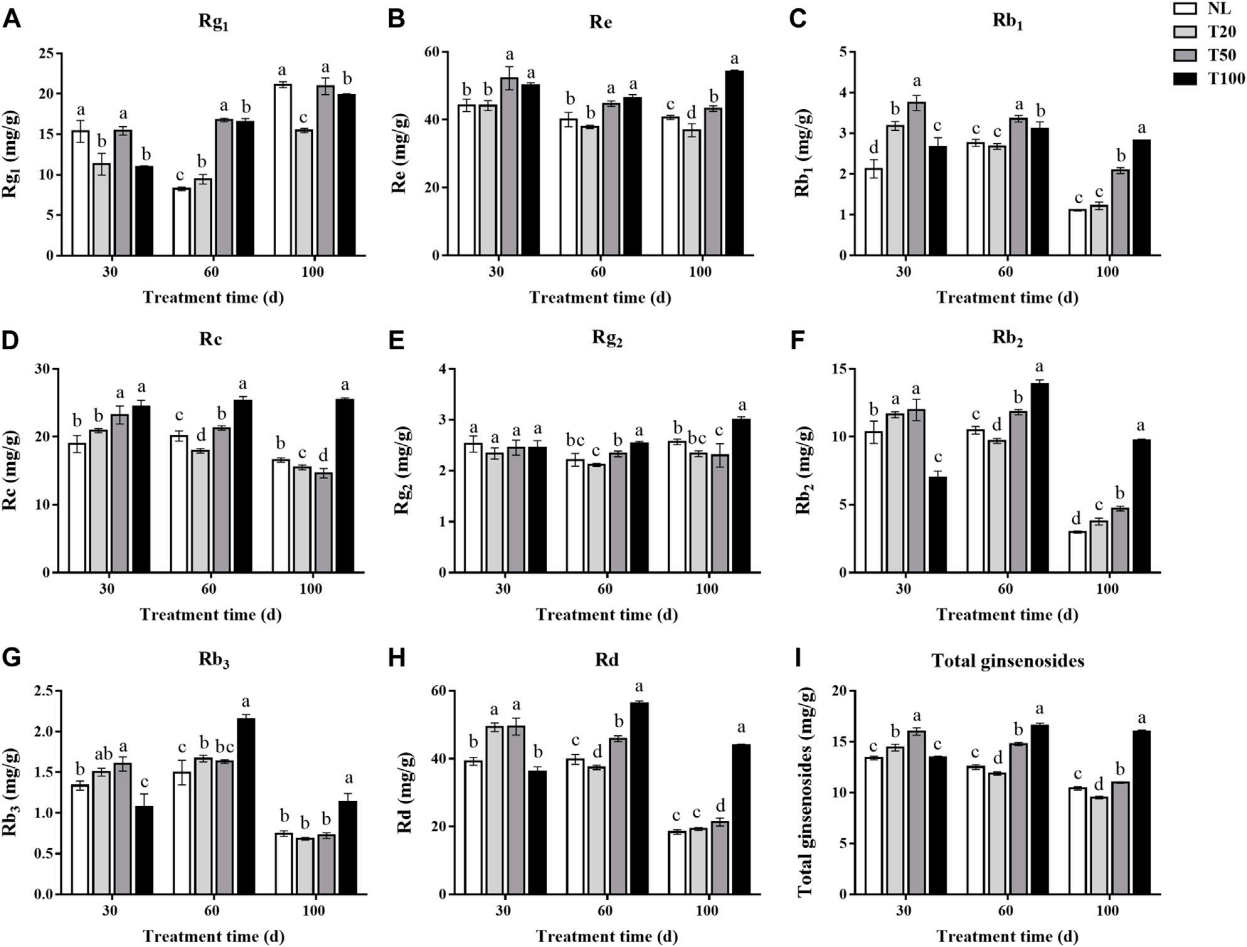
Ginsenosides content (mg/g) of *Panax ginseng* leaf under different light intensities. **(A)**, ginsenoside Rg1; **(B)**, ginsenoside Re; **(C)**, ginsenoside Rb1; **(D)**, ginsenoside Rc; **(E)**, ginsenoside Rg2; **(F)**, ginsenoside Rb2; **(G)**, ginsenoside Rb3; **(H)**, ginsenoside Rd; **(I)**, total ginsenosides NL, natural light treatment; T20, 20 µmol m^−2^·s^−1^ light intensity; T50, 50 µmol m^−2^·s^−1^ light intensity; T100, 100 µmol m^−2^·s^−1^ light intensity. The data are expressed as the mean ± standard deviation (SD) (Duncan, *p* < 0.05).

The leaf is an important medicinal component of *P. ginseng* and is rich in ginsenosides. The changes in ginsenoside contents (Rg1, Re, Rb1, Rc, Rg2, Rb2, Rb3, and Rd) in *P. ginseng* leaves under different light intensities are shown in Figure 4. After 30 days of light intensity treatment, the contents of ginsenosides Rg1, Re, Rb1, Rc, Rb2, Rb3, and Rd in the T50 treatment were substantially higher than in the T20 treatment. The content of Rg1 in the T50 treatment reached 15.43 mg/g, which was 36.38% higher than that of T20 treatment. And the content of Re reached 52.23 mg/g, which was 18.24% higher than that of T20 treatment. After 60 days of light intensity treatment, the ginsenoside content in *P. ginseng* leaves continuously increased with higher light intensity. Specifically, ginsenosides Rg1 and Rb1 reached their highest levels under the T50 treatment, whereas the other ginsenosides reached their highest levels under the T100 treatment. The content of Rg1 in the T50 treatment reached 16.76 mg/g, which was 1.77 times higher than that of T20 treatment. And the content of Re reached 44.75 mg/g, which increased 18.03% higher than that of T20 treatment. *Panax ginseng* was harvested after a 100-day light intensity treatment. The ginsenoside content of *P. ginseng* leaves still increased with increasing light intensity. Of the three light treatments, the content of each ginsenoside was highest in the T100 treatment. Ginsenosides Rg1, Re, Rb1, Rc, Rg2, Rb2, Rb3, and Rd were 1.28-, 1.47-, 2.32-, 1.64-, 1.28-, 2.59-, 1.66-, and 2.28-times higher than in the T20 treatment. The differences in the content of ginsenosides Rc, Rg2, Rb2, Rb3, and Rd between the T50 and T20 treatments were not statistically significant. As the duration of the light intensity treatment increased, the difference in saponin content between the high and low light intensity treatments became more pronounced.

**FIGURE 5:**
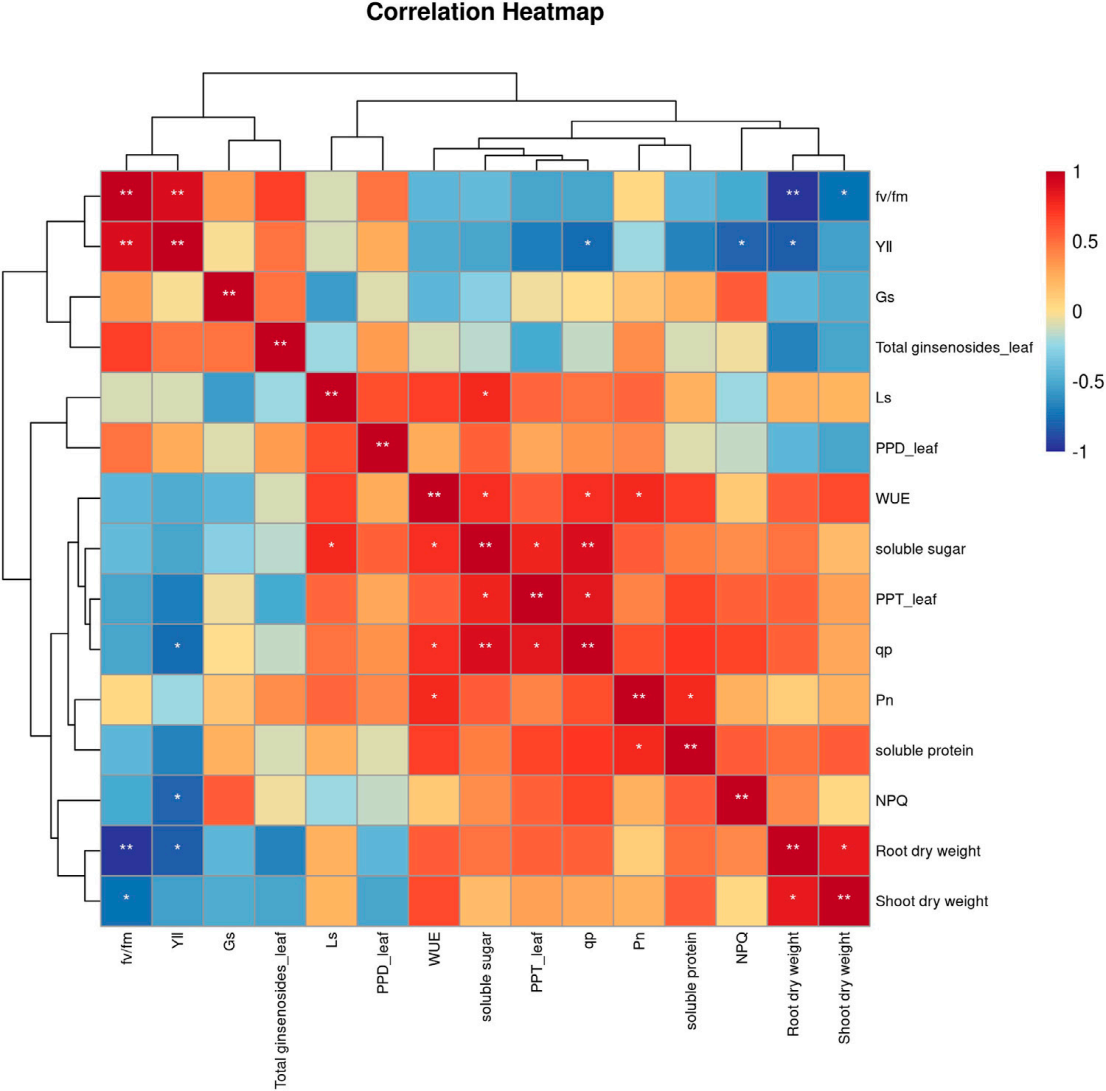
Correlation analysis between physiological indicators and ginsenosides of *Panax ginseng* under different light intensity conditions.

To elucidate how secondary metabolites in *P. ginseng* responded to various physiological and ecological indicators under different light intensities, a correlation analysis was performed between the physiological and ecological indicators and ginsenoside content (Figure 5). PPT-type ginsenosides in the leaves were positively correlated with soluble sugars and photochemical quenching coefficients. Soluble sugar content was positively correlated with water utilization and the photochemical quenching coefficient, and soluble protein was positively correlated with net photosynthetic rate. Shoot dry weight was positively correlated with root dry weight. In summary, the accumulation of dry matter in the aboveground parts of *P. ginseng* was correlated with the accumulation of dry matter in the roots, aligning with the nutrient transport pattern in the plant. Furthermore, the synthesis and accumulation of ginsenosides in different plant parts are influenced by a combination of diverse physiological and ecological indicators.

### 3.6 Metabolome data analysis

**FIGURE 6:**
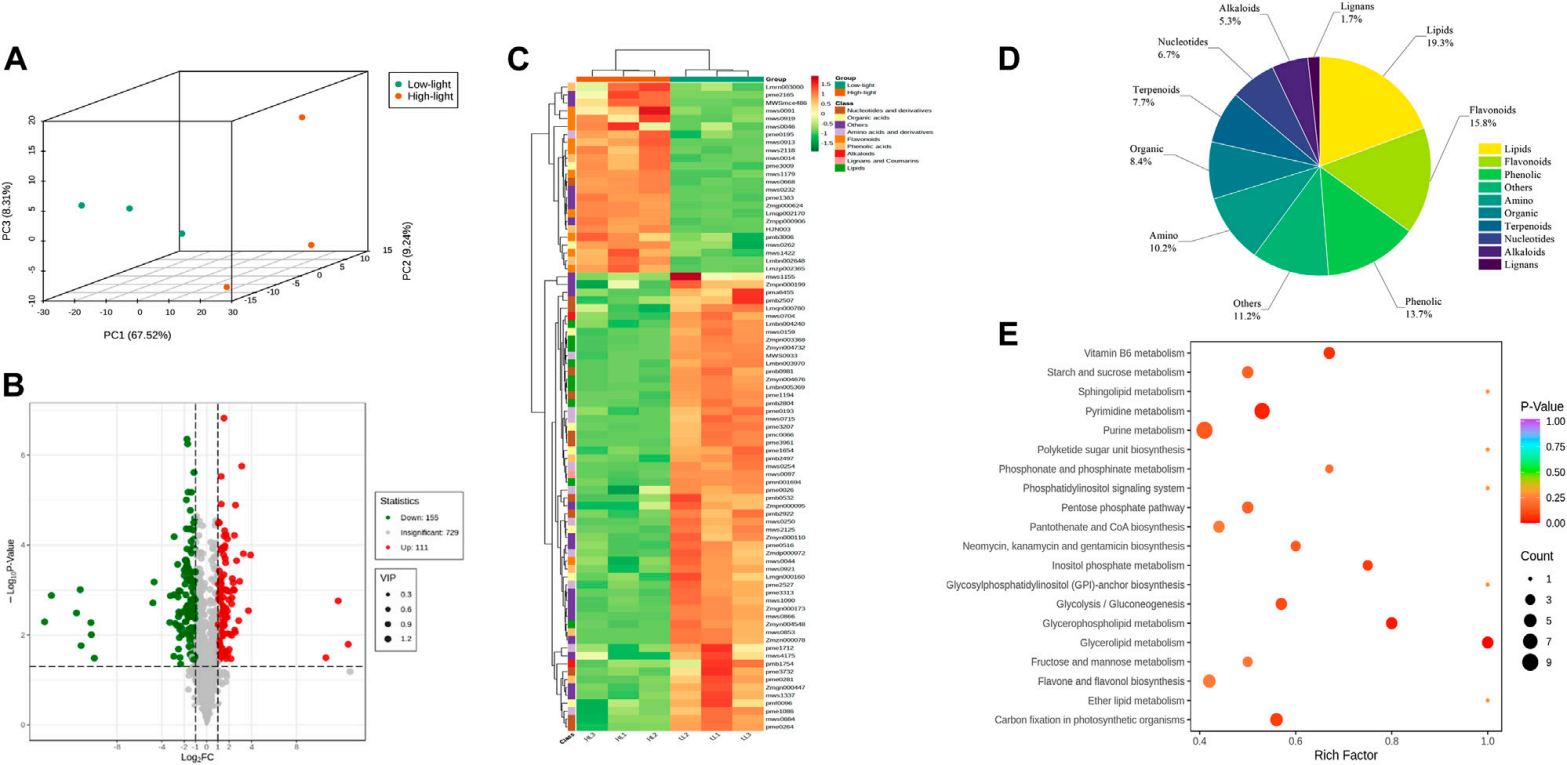
Basic metabolite information of LL and HL treatments based on Widely Targeted Metabolomics **(A)**, Principal component analysis of the metabolites identified in LL and HL treatments; **(B)**, Volcano map analysis of LL and HL treatment; **(C)**, Cluster analysis showing differentially accumulated metabolites (DAMs) upregulated and downregulated between LL and HL samples; **(D)**, Pie chart of DAMs; **(E)**, KEGG enrichment analysis of DAMs.

Widely targeted metabolomics were conducted on the T20 treatment (Low light, LL) and T100 treatment (High light, HL) to compare their global metabolic profiles. A total of 995 metabolites were identified in the ginseng leaf samples, including 12 major classes: 104 amino acids and derivatives, 147 phenolic acids, 60 nucleotides and derivatives, 126 flavonoids, 33 lignans and coumarins, 63 alkaloids, 103 terpenoids, 87 organic acids, 155 lipids, 1 tannin, 2 quinones, and 114 others. Principal component analysis of the metabolites showed a clear distinction between the two light intensity treatments with low separation between sample replicates. This indicated marked differences in the metabolites between the two treatments, and the data were reliable for subsequent analyses (Figure 6A). Both the volcano plot and heat map of the differential metabolites revealed substantial differences between *P. ginseng* samples under the two different light intensities (Figures 6B, C). Differential metabolites were screened based on the following criteria: Fold Change ≥ 2, q-value ≤ 0.5, and VIP ≥ 1. A total of 285 differential metabolites were identified, comprising 120 upregulated and 165 downregulated metabolites. The pie chart of the differential metabolites showed that there were 55 lipids, 45 flavonoids, 39 phenolics, 32 others, 29 amino acids, 24 organic compounds, 22 terpenoids, 19 nucleotides, 15 alkaloids, and 5 lignans among the identified differential metabolites (Figure 6D). The top 20 KEGG pathways with substantial enrichment were selected from the scatter plot (Figure 6E). Differential metabolites were mainly enriched in pathways such as pyrimidine metabolism, glycerolipid metabolism, Vitamin B6 metabolism, carbon fixation in photosynthetic organisms, inositol phosphate metabolism, and glycerophospholipid metabolism.

The results of differential terpene metabolites in *P. ginseng* leaves under different light intensities are presented in Supplementary Table S1. Among these metabolites, three pterpenoids were upregulated and one was downregulated, and one triterpenoid was upregulated. Additionally, 13 triterpenoid saponins were upregulated, and three were downregulated. Terpenoid biosynthesis primarily occurs via the mevalonate (MVA) and methylerythritol-4-phosphate (MEP) pathways, both starting from acetyl coenzyme A. High light intensity may enhance the accumulation of terpenoid components by potentially upregulating key enzyme genes involved upstream of the terpenoid synthesis pathway.

### 3.7 Transcriptome data analysis

**FIGURE 7:**
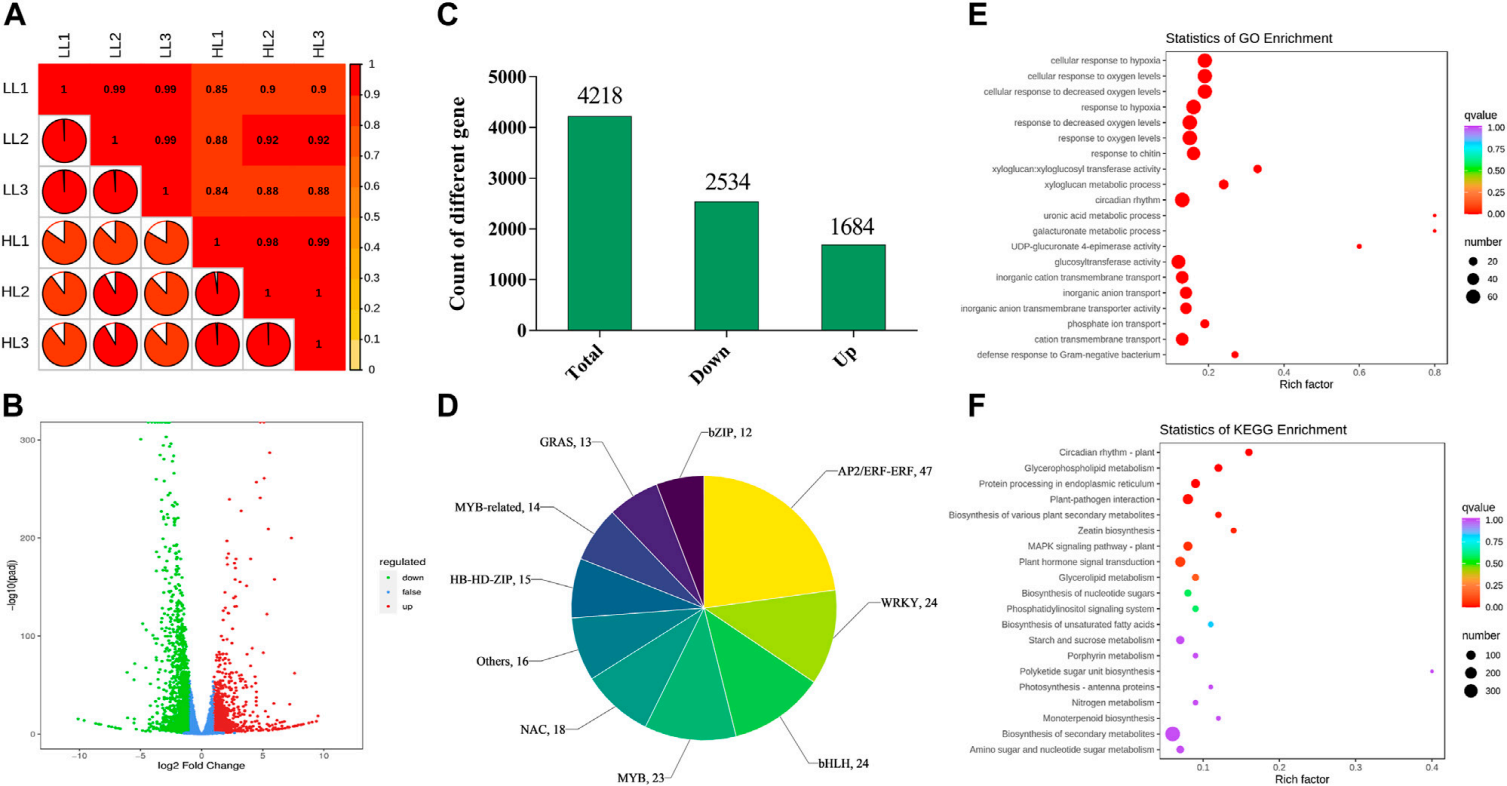
Basic genetic information of LL and HL based on transcriptomics **(A)**, Correlation analysis of LL and HL samples; **(B)**, Volcano plot of differentially expressed genes (DEGs) in LL and HL treatment; **(C)**, Number of DEGs in LL and HL treatment; **(D)**, A family of differentially expressed transcription factors in LL and HL treatment; **(E)**, GO enrichment analysis of DEGs; **(F)**, KEGG enrichment analysis of DEGs.

To explore the molecular mechanisms by which different light intensities affect *P. ginseng*, treatments with the most prominent disparities in photosynthetic physiology and secondary metabolites (T20, LL; T100, HL) were selected for transcriptome analysis. After removing low-quality data, 271, 211, and 986 clean data points were obtained. The percentage of bases with a data quality of Q30 (sequencing accuracy of 99.9%) exceeded 90%. The GC percentages of both G and C bases ranged from 41.75% to 42.96%. The sample correlation heat map showed that the *R* ^2^ of all three biological replicates within this sample group exceeded 0.85, indicating the high quality of the biological replicates for *P. ginseng* samples (Figure 7A). The volcano plot showed a large difference between the two light intensity treatments at the transcriptional level (Figure 7B). Based on Log_2_ FC and *p* < 0.05, 4218 differentially expressed genes (DEGs) were screened. Among these DEGs, 2534 were downregulated and 1684 were upregulated following HL treatment (Figure 7C). In total, 397 differential transcription factors were annotated from the DEGs, of which 152 were upregulated and 245 were downregulated. The AP2/ERF-ERF, WRKY, bHLH, MYB, and NAC families had the highest number of annotated transcription factors (Figure 7D). A scatter plot of the genes indicated DEGs were mainly enriched in the response to decreased oxygen levels, response to oxygen levels, circadian rhythm, glucosyltransferase activity, metal ion homeostasis, response to chitin, inorganic cation transmembrane transport, and the photosystem II oxygen-evolving complex (Figure 7E). KEGG enrichment analysis showed that the DEGs were predominantly enriched in biosynthesis of secondary metabolites, plant hormone signal transduction, plant–pathogen interaction, MAPK signaling pathway, protein processing in the endoplasmic reticulum, starch and sucrose metabolism, carbon metabolism, and plant circadian rhythm (Figure 7F).

### 3.8 Analysis of functional genes under different light intensities

**FIGURE 8:**
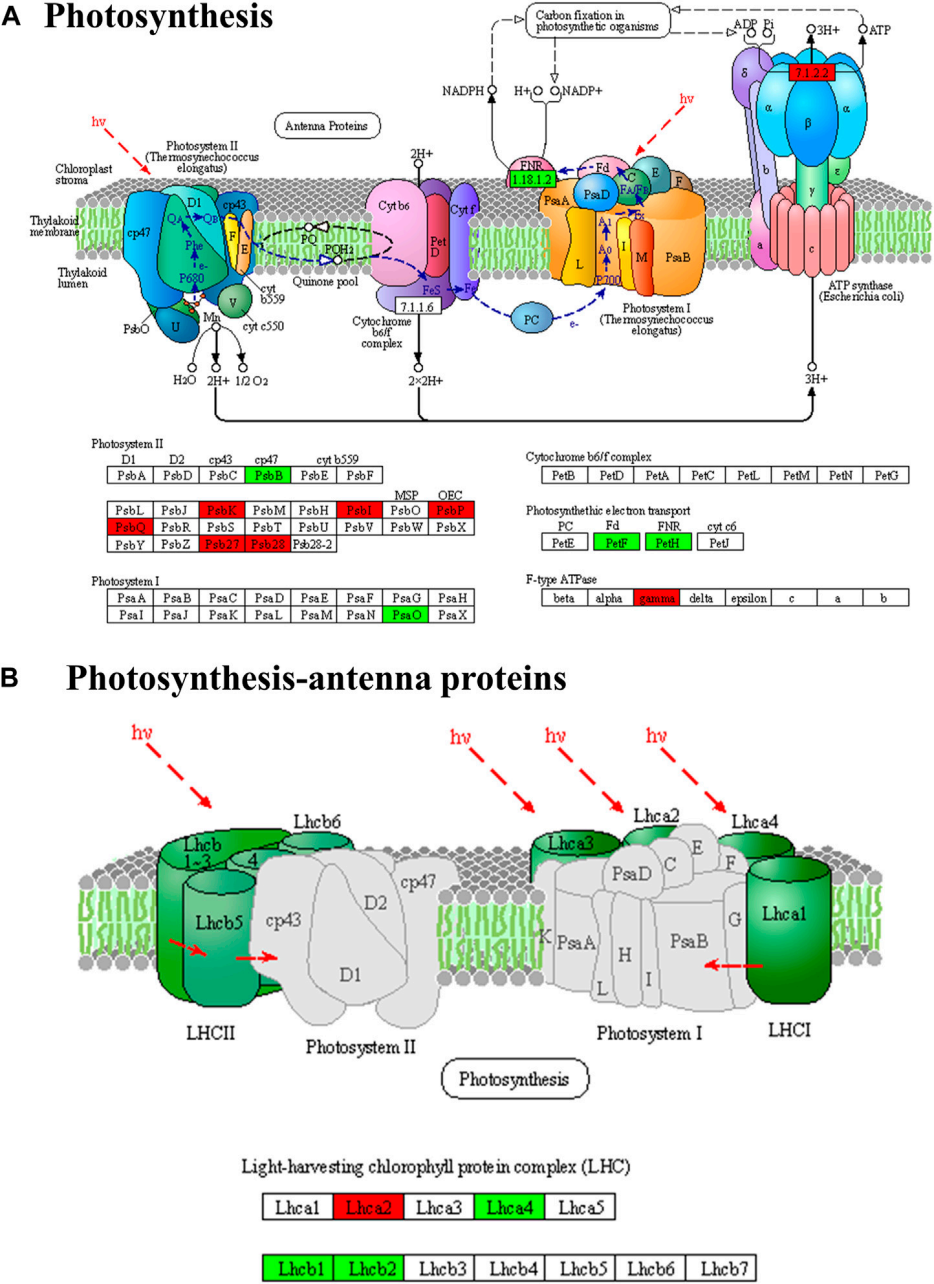
Differentially expressed genes of photosynthesis **(A)** and photosynthesis-antenna protein pathways **(B)** in *Panax ginseng* leaves. *Panax ginseng* plants were exposed to LL treatment (20 µmol m^−2^·s^−1^ light intensity), HL treatment (100 µmol m^−2^·s^−1^ light intensity).

According to the enrichment and annotation of transcriptome gene functions, differential gene expression was observed in various pathways in *P. ginseng* samples under different light intensities. These pathways included photosynthetic and photosynthesis antenna proteins, carbon fixation in photosynthetic organisms, citrate cycle pathways, terpene metabolism, and light signal transduction. A total of 26 DEGs were identified in the photosynthetic pathway, of which 16 were upregulated and eight were downregulated (Figure 8). Specifically, in photosystem I, two genes encoding reaction center proteins were downregulated and four were upregulated, whereas in photosystem II, 14 genes encoding reaction center proteins were upregulated and two were downregulated. Additionally, two ATP synthase genes were upregulated, and four DEGs were downregulated during photosynthetic electron transport.

**FIGURE 9:**
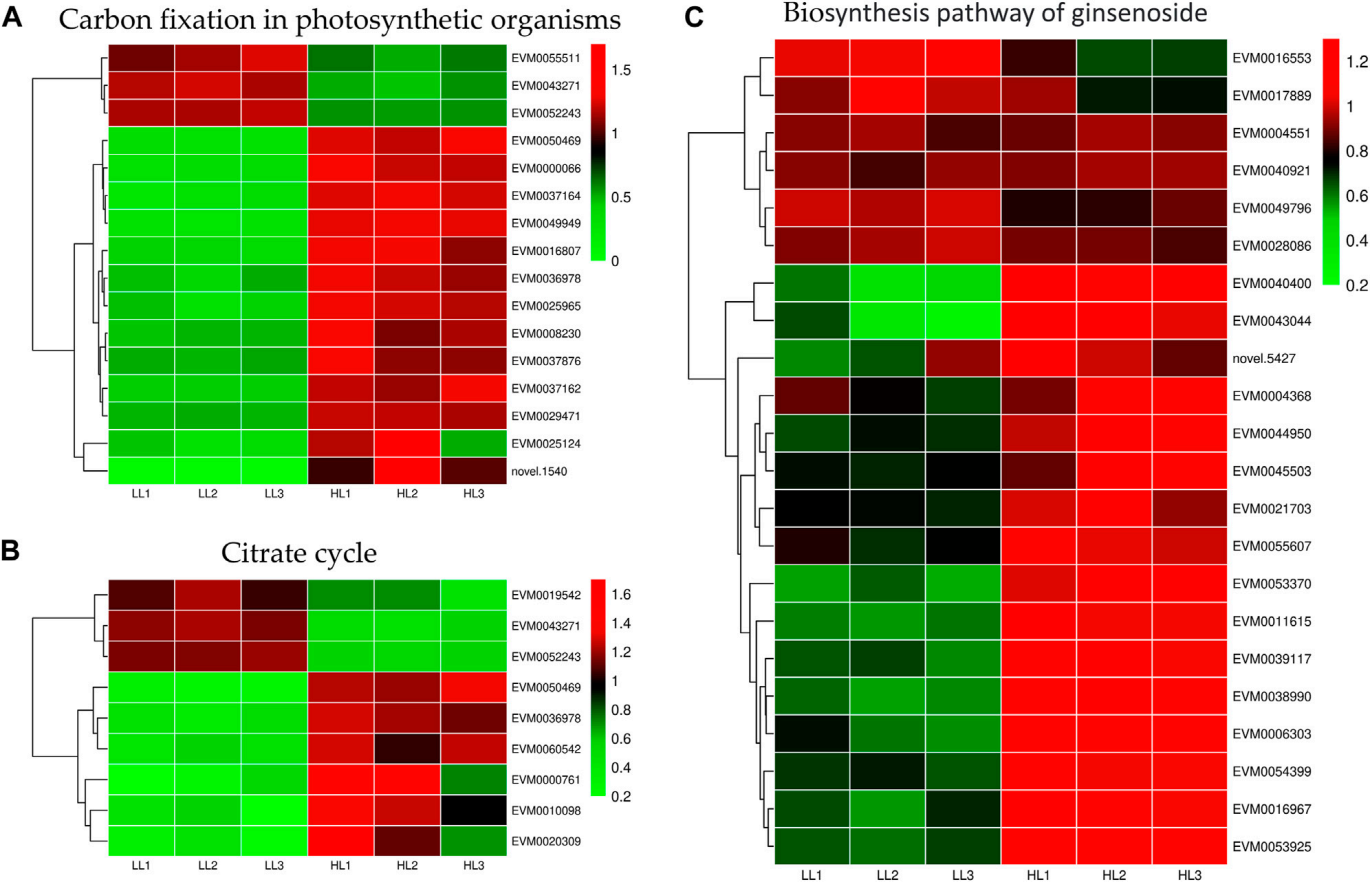
Differentially expressed genes in carbon fixation in photosynthetic organisms **(A)**, citrate cycle **(B)**, and biosynthesis of ginsenosides **(C)**.

Light intensity affected gene expression in the carbon fixation of photosynthetic organisms and the TCA cycle pathways (Figures 9A, B). We identified 16 differentially expressed genes in the carbon fixation pathway of photosynthetic organisms, with 13 upregulated and 3 downregulated genes. Specifically, under HL treatment, the following gene expressions were upregulated: malate dehydrogenase (*EVM0036978* and *EVM0050469*), glyceraldehyde-3-phosphate dehydrogenase (*EVM0037162*, *EVM0025124*, and *EVM0008230*), transketolase (*EVM0029471*), phosphoenolpyruvate carboxylase (*EVM0037164*, *EVM0049949*, *EVM0037876*, *EVM0000066*, and *EVM0016807*), and ribulose bisphosphate carboxylase small subunit (*EVM0025965*). We identified nine DEGs in the TCA cycle pathway, with six upregulated and three downregulated genes. Specifically, under high-intensity light, malate dehydrogenase (*EVM0036978* and *EVM0050469*), pyruvate dehydrogenase E1 component subunit alpha-3 (*EVM0060542*), pyruvate dehydrogenase E1 component subunit alpha (*EVM0000761*), and dihydrolipoyl dehydrogenase (*EVM0010098* and *EVM0020309*) were upregulated. Therefore, high light intensity promoted energy metabolism in *P. ginseng*, which potentially contributed to an increase in *P. ginseng* biomass.

**FIGURE 10:**
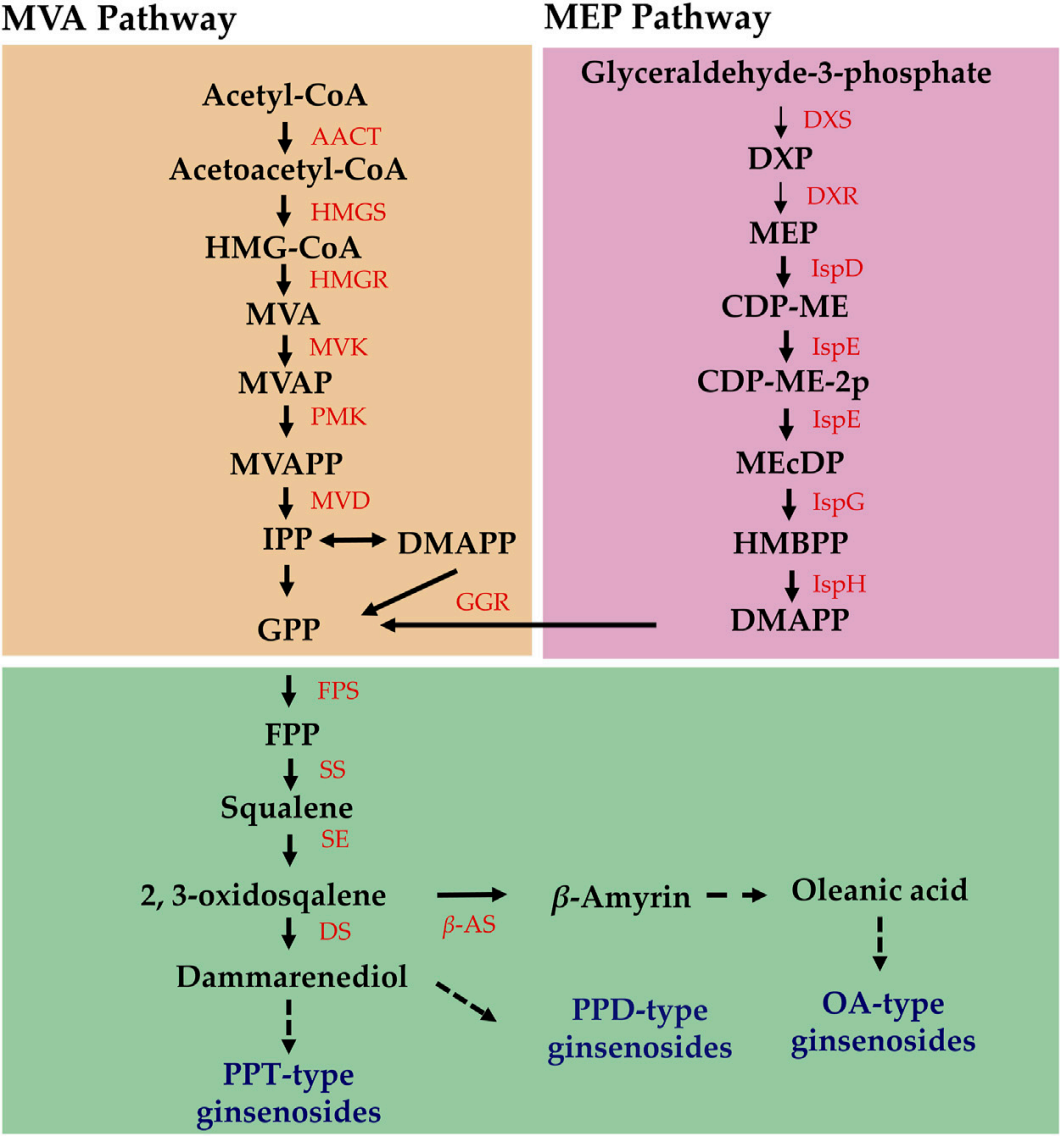
Ginsenoside biosynthetic pathway in *Panax ginseng*.

Elucidating the biosynthetic pathways of medicinal ingredients is crucial for the quality control of *P. ginseng*. The main active ingredient in *P. ginseng* is ginsenoside, a triterpenoid compound, and there is a solid foundation for studying its synthetic pathways. Metabolomic data demonstrated an increase in terpene content after HL treatment, and the results of HPLC in *P. ginseng* samples confirmed that HL treatment promoted the accumulation of ginsenosides. To investigate the mechanism of terpene synthesis under different light intensities, the key enzyme genes that have been reported to play important roles in the ginsenoside synthesis pathway were screened from the transcriptomic data and a heat map of gene expression was plotted (Figure 9C). Ginsenoside biosynthetic pathway in *P. ginseng* was shown in Figure 10. Transcriptomic data indicated that two 3-hydroxy-3-methylglutaryl coenzyme A reductases (*HMGR, EVM0039117 and EVM0044950*) were upregulated under high-light intensity. Additionally, two farnesyl pyrophosphate synthases (*FPS, novel.5427 and EVM0016553*) displayed divergent expression patterns, with one being upregulated and the other being downregulated. The expression of the three squalene synthases (*SS, EVM0011615, EVM0040400 and EVM0016967*) were upregulated. Two squalene cyclooxygenases (*SE, EVM0053925 and EVM0054399*) were upregulated. Expression levels of the two dammarendiol-II synthases (*DS, EVM0017889 and EVM0004551*) were not substantially different. Cytochrome P450 (CYP) and glycosyltransferase are the downstream genes involved in ginsenoside biosynthesis. We found that the two *CYP716A47* (EVM0049796 and EVM0028086)genes were similar in expression. The expression of two *CYP716A53v2 (EVM0004551 and EVM0043044)*, two *CYP716A52v2 (EVM0021703 and EVM0040921)*, two *UGT74AE (EVM0045503 and EVM0055607)*, one *PgUGT1(EVM0053370)*, and two *UGTPg45 (EVM0006303 and EVM0004368)* genes was upregulated. In conclusion, the expression of key enzyme synthesis genes, including HMGR, SS, *CYP716A53v2*, *UGT74AE*, *PgUGT1*, and UGTPg45, promoted the synthesis of terpenoids and saponins.

**FIGURE 11:**
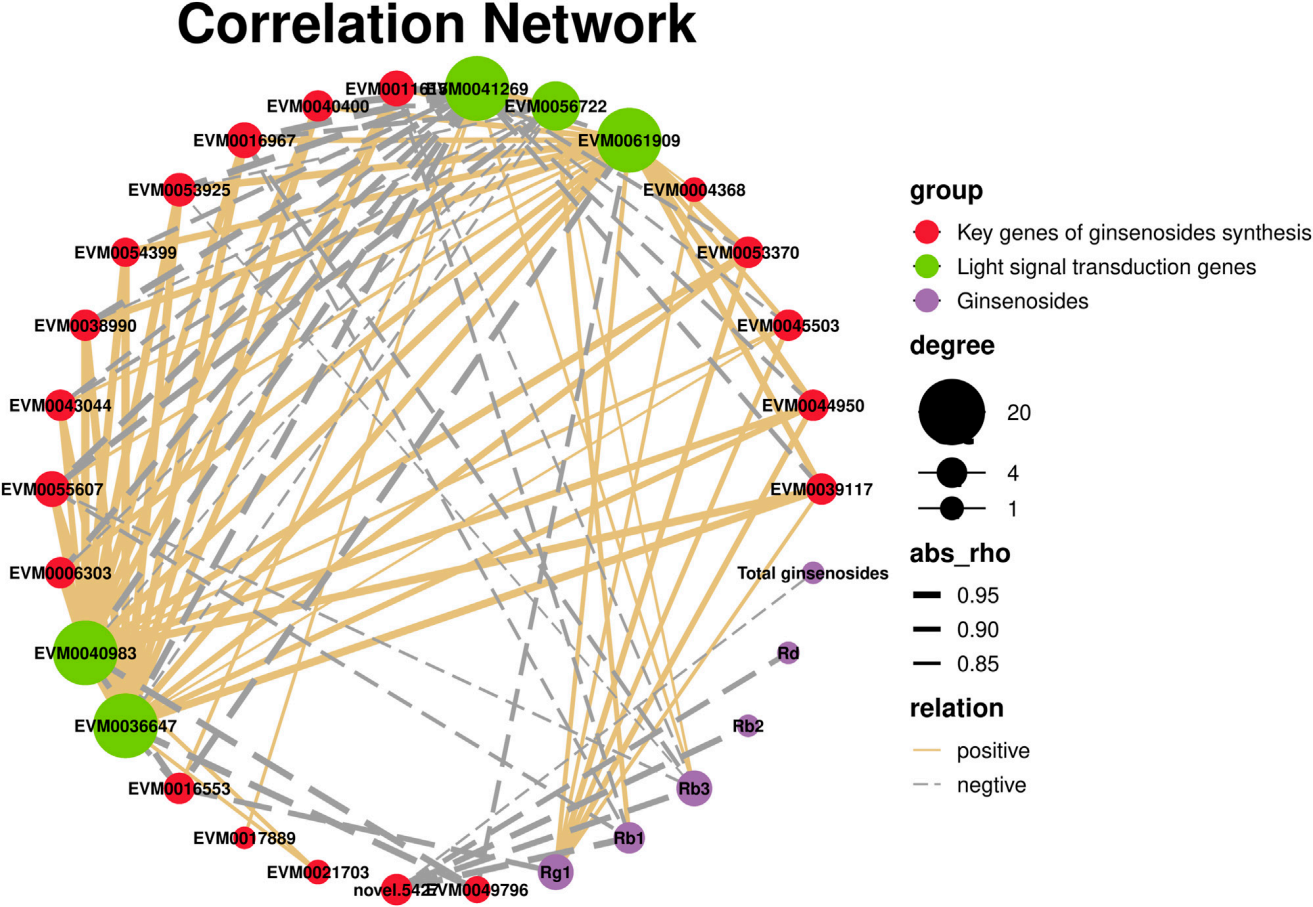
Correlation network of light signaling genes, key genes of ginsenosides synthesis, and ginsenosides.

Plants have a complex light signal transduction system that regulates a series of life processes by recognizing light signals of different wavelengths and transmitting them downwards through photoreceptors. To explore the response of light signal transduction pathways to varying light intensities, we investigated the photoreceptor and light signal transduction genes in the transcriptomic data (Table 2). We found that cryptochrome-2 (*EVM0041269*, *CRY*), an E3 ubiquitin ligase (*EVM0061909*, *COP1*), three transcription factors (*PIF*, *EVM0040983* and *EVM0036647*), and one transcription factor (*HY5*, *EVM0056722*) were upregulated following HL treatment. Correlation network analysis was conducted to investigate the relationship between light signaling genes, key genes involved in ginsenoside synthesis, and ginsenosides (Figure 11). The results showed positive correlations between ginsenosides Rg1, Rb1, and Rb3 and multiple key ginsenoside synthesis genes. Furthermore, the transcription factor, PIF, was co-expressed with several key enzymes involved in ginsenoside synthesis. Based on these findings, we hypothesized that PIF participates in ginsenoside biosynthesis under different light intensities.

**TABLE 2:** Transcript levels of differential genes related to light signal transduction.

Gene ID	*p*-value	log_2_FC	Regulated	Annotation
EVM0061909	8.16705E-09	1.63	up	E3 ubiquitin-protein ligase COP1
EVM0041269	5.67899E-08	−1.69	down	Cryptochrome-2
EVM0040983	2.08118E-86	1.43	up	Transcription factor PIF3
EVM0036647	5.46924E-56	1.53	up	Transcription factor PIF3
EVM0056722	2.14736E-05	−1.27	down	Transcription factor HY5

FC, fold change.

## 4 Discussion

### 4.1 Light intensity affects growth of P. ginseng

Light intensity affects the structure and function of plant photosynthetic units, as well as the absorption and utilization of light energy (Modarelli et al., 2022). Plant growth and development require an appropriate lighting environment. In general, high light intensity results in decreased leaf area, leaf yellowing, and leaf wilting, whereas low light intensity promotes increased plant height, weak stems, and poor growth (Morelli et al., 2021). In the current study, three light intensity gradients were selected, ranging from the light saturation point to the compensation point for *P. ginseng*. The dry weight of *P. ginseng* increased under higher light intensities. By the end of the harvest period, the dry weight of *P. ginseng* roots reached its highest value under the T100 treatment, which was 14.00% higher than that under the T20 treatment. Shoot dry weight was highest under the T50 treatment (Figure 1). Investigating the optimal light intensity for *P. ginseng* cultivation under artificial light is important for the advancement of plant factory cultivation in *P. ginseng*. The increase in *P. ginseng* biomass under the higher light intensity treatment was attributed to the impact of light intensity on plant photosynthetic characteristics (Keller et al., 2022). Photosynthesis accounts for over 90% of the dry matter accumulation in plants, and crop yield is frequently dependent on the photosynthetic rate. Higher light intensity in a suitable range stimulates transpiration in plants, enabling them to absorb more water and nutrients to maintain an optimal metabolic balance and promote biomass accumulation in the lower part of the soil (Gao P. et al., 2022). In general, higher light intensity promotes the accumulation of dry matter in *P. ginseng* plants. Aboveground and belowground dry weight accumulation in ginseng plants responded differently to medium and high light intensities at different stages of *P. ginseng* growth and development.

### 4.2 Effects of light intensities on physiological and ecological changes of P. ginseng

Photosynthetic parameters directly reflect the capacity of a plant to absorb carbon dioxide and release oxygen. The Pn of *P. ginseng* increased with higher light intensity after 30 days of light treatment and was highest under the T100 treatment, which was 1.15-times higher than that of the T20 treatment (Figure 2A). At this stage, there were no marked differences in stomatal conductance or stomatal limitation values among the treatments (Figures 2B, C), indicating that non-stomatal factors limit the photosynthetic rate of *P. ginseng* under low light intensity (Sakoda et al., 2021). This limitation may be attributed to the decreased activity of key enzymes in the carbon assimilation process or weakened photochemical reaction performance under low light intensity. Moreover, after 100 days of light treatment, the Pn of under the T100 treatment was lower than that under the T50 treatment. Additionally, stomatal conductance was the highest under the T50 treatment at this stage, whereas the stomatal limiting value increased substantially under the T100 treatment. Therefore, the factors that limit the increase in photosynthesis rate under higher light intensity at this stage were primarily associated with stomatal factors, resulting in a decrease in intercellular CO_2_ concentration and the inhibition of photosynthesis (Hutmacher and Krieg, 1983; Ghorbanzadeh et al., 2021).

Chlorophyll fluorescence technology provides a rapid, effective, and non-invasive method for the determination of photosynthetic activity in photosystem II (PSII), which reveals differences in excitation energy allocation in the photosystem and heat dissipation mechanisms in response to environmental changes in plants (Oukarroum, 2016). Maximum photochemical efficiency (Fv/Fm) reflects the conversion efficiency of primary light energy in photosystem II and serves as an indicator of plant stress (Maxwell and Johnson, 2000). There were no marked differences in Fv/Fm among the treatments, suggesting that *P. ginseng* did not experience light stress when light intensity was increased within a suitable range (Figure 2E). The actual photochemical efficiency (φPSII) represents the quantum yield of PSII photochemistry. This indicates the proportion of excitation energy utilized in photochemical pathways relative to the total excitation energy absorbed by PSII (Zheng et al., 2021). This parameter is a crucial indicator of the photosynthetic capacity of plants. The φPSII followed the order of T20 > T50 > T100 (Figure 2F), indicating an increase in the surplus of quantum light yield for photochemical pathways as the light intensity increased. Simultaneously, the non-photochemical quenching coefficient (NPQ) increased at higher light intensities in *P. ginseng* (Figure 2H). This indicates that the excessive light energy captured by the leaves did not enter the electron transport chain but dissipated as heat under high light intensity. Thermal dissipation is a protective mechanism in plants that prevents the photoinhibition of leaves caused by excessive light energy (Goss and Lepetit, 2015).

The contents of soluble sugars and soluble proteins increased in response to higher light intensity (Figures 3A, B), which might be because light intensity affected the levels of carbon and nitrogen metabolism in *P. ginseng*. Based on the photosynthetic response of *P. ginseng* to different light intensity gradients, it is recommended that high light intensity be provided during the early growth stage and be gradually reduced to medium intensity during the fruiting stage. Developing a light control scheme that aligns with the requirements of light intensity in *P. ginseng* at different growth stages is beneficial for maximizing the yield and increasing the energy efficiency in artificial light cultivation mode of *P. ginseng*.

### 4.3 Light intensity affects content of secondary metabolites in P. ginseng

Medicinal plants can be categorized into three types based on their light intensity requirements: sun-loving, shade-loving, and intermediate (Ko et al., 2020). Light intensity strongly influences the synthesis and accumulation of secondary metabolites in medicinal plants (Xu et al., 2020), which in turn leads to variations in the type and composition of these metabolites, resulting in differences in the pharmacological activity of the herbs. Previous studies have demonstrated the marked effect of different light intensities on the accumulation of flavonoids (Wang et al., 2022), alkaloids (Hoft et al., 1996), and other components in medicinal plants (Zhang S. et al., 2021). The results of this study demonstrate that light intensity influences ginsenoside accumulation. Monomeric saponin and total ginsenoside content were substantially lower in the T20 and T50 treatments than in the T100 treatment (Figure 4). The total ginsenoside content in *P. ginseng* leaves reached 16.01% after 100 days of high light intensity treatment, which was 53.69%, 68.32%, and 45.55% higher than that of the NL, T20, and T50 treatments, respectively. Therefore, considering the accumulation of secondary metabolites, medium-intensity light treatment (T50) was found to be more favorable for ginsenoside accumulation in the leaves during the early stages of *P. ginseng* growth and development. In contrast, the accumulation of ginsenosides in the leaves reached its highest value under high light intensity (T100) during the middle and late stages of *P. ginseng* growth.

### 4.4 Leaf transcriptome and metabolome responses to different light intensities

According to the growth results, photosynthetic physiology, soluble sugar, soluble protein content, and ginsenoside content are adjusted in *P. ginseng* under different light intensities. Differential responses in physiological processes and secondary metabolism to different light intensities were observed. To investigate the underlying reasons for these changes, transcriptomic and metabolomic techniques were used to elucidate the physiological and secondary metabolic mechanisms of *P. ginseng* under different light intensities. Transcriptome techniques enable the study of gene transcription across all transcripts in a specific tissue or cell at a particular stage, providing comprehensive insights into the laws of transcriptional regulation. In contrast, metabolomic techniques offer feedback on gene expression and protein function (Behera et al., 2021). To date, no studies have investigated the impact of light intensity on *P. ginseng* using transcriptomics and metabolomics. The integrated data obtained from transcriptomics and metabolomics provide a novel understanding of the responses to light intensity in *P. ginseng*. The two light intensities caused substantial differences at the transcriptional and metabolic levels. Moreover, different light intensities influenced terpene metabolism, photosynthesis, antenna proteins, carbon fixation pathways, and the TCA cycle in *P. ginseng*.

Photosynthesis in plants involves photosystem I (PSI) and photosystem II (PSII), as well as ATP synthase (F-ATPase) and cytochrome (cyt) b6f complexes (Tan et al., 2020). We identified 26 DEGs in the photosynthetic pathway; 16 DEGs were specifically associated with PSII. Of these, 14 genes encoding reaction center proteins (PSBI, PSBK, PsbP, PsbQ, Psb27, and PSB28) exhibited higher expression levels under high light intensity than under low light intensity (Supplementary Table S2). PSII is comprised of various protein subunits, including PsbA, PsbO, PsbU, PsbV, and PsbX (Cardona et al., 2019). These proteins, which encode reaction-center complexes, play crucial roles in the formation of photoreaction center structures. Changes in natural light intensity induce structural and functional adjustments in photosynthesis to adapt to different light levels. This phenomenon is commonly known as photoacclimation in plants (Ben-Sheleg and Vonshak, 2022). The current study found that *P. ginseng* exhibited high light acclimation characteristics. This also explains the upregulation of genes involved in the synthesis of the reaction center proteins of PSII and ATP synthase. Consequently, both the photosynthetic rate and photochemical quenching coefficient of ginseng leaves reached their highest values at high light intensities.

The plant photosystem comprises light-harvesting complexes and photosynthetic reaction centers. The light-harvesting complex consists of proteins and pigments, which capture light energy and transfer it to the photosynthetic reaction center. Upon photon absorption, the energy of a pigment molecule is rapidly transferred through resonance energy transfer until it reaches the photosynthetic reaction center. The response of photosynthetic apparatus to light intensity has been extensively investigated in model plants and crops. To adapt to low light intensities, leaves usually decrease their allocation of resources to carbon assimilation-related enzymes. Conversely, leaves allocate more resources to the light-capture apparatus, enhancing the efficiency of light utilization under low-light conditions (Poorter et al., 2019). The current study identified four genes encoding antenna proteins that were upregulated under low light intensity, whereas two genes were downregulated (Supplementary Table S3). The upregulation of Lhcb1, Lhcb1, and Lhcb2 at low light intensities is the main constituent of the photosystem light-harvesting protein complex (Hippler and Nelson, 2021). To adapt to low light intensities, *P. ginseng* increased the number of light-harvesting complexes and the antennae/reaction center ratio. Although the photosynthetic efficiency of leaves is reduced under low light intensity, these adaptations maximize the interception and utilization of light energy. In conclusion, photoadaptation occurs in *P. ginseng* under both high and low light intensities. Photoacclimation allows wild plants to optimize resource utilization in complex natural environments, thereby achieving a balance between survival and reproduction. In *P. ginseng* cultivation, maximizing yield is crucial for improving economic value. Overcoming the limitations of photosystem photoadaptation and selecting and breeding superior varieties are essential strategies in the development of *P. ginseng* cultivation systems and industry.

Various light intensities had marked effects on the energy metabolism of *P. ginseng*. Notably, gene expression in carbon fixation of photosynthetic organisms and the TCA cycle pathway was upregulated (Figures 9A, B). Carbon fixation in photosynthetic organisms occurs during photosynthetic reactions in the dark. ATP and NADPH produced in the light reaction are utilized to convert CO_2_ into stable carbohydrates, contributing to the synthesis of plant organic matter. Sixteen DEGs were identified in this pathway, with 13 upregulated and three downregulated genes. Malate dehydrogenase (MDH) facilitates the conversion of malate and oxaloacetate in the C4 dicarboxylic acid cycle, ensuring dynamic equilibrium (Venkat et al., 2017). Glyceraldehyde-3-phosphate dehydrogenase (GAPDH) catalyzes the conversion of glyceraldehyde-3-phosphate to 1,3-diphosphoglycerate (Wei et al., 2022). Transketolase (TKL) catalyzes the reaction of glyceraldehyde 3-phosphate and fructose 6-phosphate to produce xylulose-5-phosphate and erythrose-4-phosphate (Huo et al., 2018). In contrast, phosphoenolpyruvate carboxylase (PEPC) is responsible for the initial fixation of CO_2_ and plays a crucial role in carbon assimilation (Feria et al., 2022). Gene expression of *MDH*, *GAPDH*, *TKL*, and *PEPC* was markedly upregulated under high light treatment (Supplementary Table S4). In the TCA cycle pathway, the gene expression of pyruvate dehydrogenase E1 component subunit alpha-3, pyruvate dehydrogenase E1 component subunit alpha, and dihydrolipoyl dehydrogenase showed the same trend (Supplementary Table S5). In the present study, high light intensity enhanced the energy metabolism of *P.* ginseng, leading to a positive impact on biomass. Energy allocation in *P. ginseng* changes with different light intensities, maintaining a balance between survival and growth.

Analysis of the biosynthetic pathways of the pharmacologically active components is essential for ensuring the quality of *P. ginseng*. Ginsenosides and triterpenoids are the primary pharmacologically active components of *P. ginseng*. Previous studies have provided a foundation for investigating the ginsenoside synthetic pathway of ginsenoside (Hou et al., 2021). In the current study, DEGs involved in ginsenoside synthesis using transcriptome data were identified. *HMGR*, *FPS*, *SS*, *SE*, *CYP716A53v2*, *UGT74AE*, *PgUGT1*, and *UGTPg45* were upregulated under high light intensity. It is hypothesized that the increased expression of genes such as *HMGR* and *FPS* in response to high light intensity leads to a marked increase in ginsenoside content. Analysis of metabolome data indicated marked increases in terpene metabolites under high light intensity. Of these, three sesquiterpenes were upregulated and one was downregulated. Additionally, one triterpene was upregulated, 13 triterpene saponins were upregulated, and three were downregulated. Therefore, high light intensity not only enhanced the synthesis of triterpenoid saponins but also promoted the accumulation of other terpenoid components in *P. ginseng* leaves. Terpenoid metabolism begins with acetyl coenzyme A, which is further synthesized through the mevalonate (MVA) pathway and methylerythritol-4-phosphate (MEP) pathways (Lipko et al., 2023). As shown in the transcriptome data, the expression levels of *HMGR*, *FPS*, and other genes were upregulated under high light intensity. Based on this, we hypothesized that high light intensity treatment increases the expression of key enzyme genes involved upstream of the terpene synthesis pathway, thereby promoting terpene synthesis. Furthermore, the terpene skeleton, consisting of carbon, and carbon metabolism is enhanced under high light intensity, which may provide substrates to promote terpene synthesis.

Plants have a complex light signal transduction system that regulates various life processes by identifying light signals of different wavelengths and transmitting them downward through photoreceptors. Kumari *et al.* (Kumari et al., 2019)demonstrated that light intensity influences the expression of genes involved in light-, hormone-, and clock-regulated pathways, including *PIF4*, *EPR1*, *COL3*, *CIP1*, and *TOC1*. These genes were important in root development. By analyzing the transcriptome data of the different light intensity treatments, three light signal transduction factors were identified: PIF (EVM0040983 and EVM0036647) and HY5 (EVM0056722). PIFs, members of the bHLH family of transcription factors, are actively involved in the light signaling pathway of plants. In *Arabidopsis*, PIF3 regulates anthocyanin synthesis by binding to the G-box element in the promoter region of anthocyanin biosynthesis genes (Ni et al., 1998). The correlation network analysis showed that there were correlations between PIF and several key enzyme genes of ginsenoside synthesis. Based on these findings, we hypothesized that expression of PIF may directly be involved in regulating the key enzyme genes of ginsenoside synthesis under high-intensity light. Alternatively, they may indirectly promote ginsenoside biosynthesis by regulating expression of other genes.

## 4 Conclusion

Our study revealed that higher light intensity positively influenced the yield, photosynthesis, and accumulation of polysaccharides, terpenoids, and ginsenosides in *P. ginseng*. The suitable light intensity range for *P. ginseng* growth was 50–100 µmol m^−2^·s^−1^.100 µmol m^−2^·s^−1^ was conducive to quality formation of *P. ginseng* leaves. Furthermore, light intensity affects the expression of genes associated with photosynthesis, photosynthesis antenna proteins, carbon fixation in photosynthetic organisms, and the TCA cycle pathway. The T100 treatment resulted in substantial adaptation to strong light. The expression of genes encoding photosystem II-reaction center proteins was upregulated, which increased photosynthetic activity and enhanced the expression of genes involved in photosynthetic carbon and energy metabolism in *P. ginseng*. Moreover, the expression of antenna protein synthesis genes was upregulated under the T20 light intensity treatment, which enhanced the light-capturing capacity of the ginseng leaves. Strong light intensity promoted the accumulation of terpenoid secondary metabolites in *P. ginseng* by upregulating gene expression in its biosynthetic pathways. Based on transcriptomics and metabolomics, this study provides new insights into the response mechanisms to different light intensities in *P. ginseng*. This study is of great significance in promoting the technological innovation of *P. ginseng* cultivation and the application of modern engineering technology in the field of ginseng cultivation.

## Data Availability

The datasets presented in this study can be found in online repositories. The names of the repository/repositories and accession number(s) can be found in the article/Supplementary Material.
